# MiR-7a is an important mediator in Fas-associated protein with death domain (FADD)-regulated expression of focal adhesion kinase (FAK)

**DOI:** 10.18632/oncotarget.9838

**Published:** 2016-06-06

**Authors:** Yingting Liu, Hongen Cui, Xianjie Huang, Bo Zhu, Shengwen Guan, Wei Cheng, Yueyang Lai, Xiaoxin Zhang, Zi-Chun Hua

**Affiliations:** ^1^ The State Key Laboratory of Pharmaceutical Biotechnology, School of Life Sciences and School of Stomatology, Affiliated Stomatological Hospital, Nanjing University, Nanjing, 210032, China; ^2^ Changzhou High-Tech Research Institute of Nanjing University and Jiangsu TargetPharma Laboratories Inc., Changzhou, 213164, China; ^3^ The State Key Lab of Natural Medicines, China Pharmaceutical University, Nanjing, 210017, China; ^4^ The State Key Laboratory of Bioelectronics, Southeast University, Nanjing, 210008, China

**Keywords:** FADD, FAK, miR-7a, melanoma, migration

## Abstract

Fas-associated protein with death domain (FADD), a classical adaptor protein mediating apoptotic stimuli-induced cell death, has been reported to engage in several non-apoptotic processes such as T cell and cardiac development and tumorigenesis. Recently, there are several reports about the FADD's involvement in cell migration, however the underlying mechanism remains elusive. Here, we present a new finding that FADD could regulate the expression of FAK, a non-receptor protein tyrosine kinase overexpressed in many cancers, and played an important role in cell migration in murine MEF and melanoma cells with different metastatic potential, B16F10 and B16F1. Moreover, miR-7a, a tumor suppressor which prohibits cell migration and invasion, was up-regulated in FADD-deficient cells. And FAK was verified to be the direct target gene of miR-7a in B16F10 cells. Furthermore, we demonstrate that miR-7a was a necessary mediator in FADD-regulated FAK expression. In contrast to its classical apoptotic role, FADD interference could reduce the rate of cell migration, which could be rescued by inhibiting miR-7a expression. Taken together, our data provide a novel explanation regarding how FADD regulates cell migration in murine melanoma cells.

## INTRODUCTION

According to the World Health Organization (WHO), in every three cancers diagnosed, there is one skin cancer. Malignant melanoma accounts for nearly 80% of deaths caused by all skin cancers despite its low occurrence compared to its non-melanoma counterparts [1, 2]. Once becoming metastatic, melanoma is associated with poor prognosis and highly intractable to both radiotherapy and chemotherapy, which causes the five-year survival rate of most patients to drop to only 14% [3, 4]. Clinical studies show that melanoma could easily spread to other parts of body, such as skin, liver, lung and brain [5]. The lack of effective therapies pushed us to further explore the complicated genetic networks involved in melanoma metastasis and seek new therapeutic strategies to improve clinical outcomes.

Fas-associated protein with death domain (FADD) is originally identified as an essential adaptor which couples death receptors with caspase-8 death signals in death receptor-mediated apoptosis [6–8]. FADD has also been demonstrated to play a role in non-apoptotic processes. FADD-deficient mice died *in utero* about 10 days in gestation, suggesting essential role of FADD in embryogenesis [9, 10]. FADD knockout lymphocytes are blocked at the DN3 stage during T cell maturation and have been reported to have impaired proliferation [11]. Recently, FADD has also been implicated in tumorigenesis and is frequently amplified in many cancer cells, acting as a biomarker [12–14]. In head and neck squamous cell carcinoma, FADD, DR5 and caspase-8 have been reported to be associated with tumor growth and metastasis [15, 16]. However, the mechanism of FADD in tumorigenesis and metastasis remains unknown and requires further investigation.

Focal adhesion kinase (FAK), a 125kDa non-receptor protein tyrosine kinase first isolated from chicken and mouse, is an important mediator of extracellular matrix integrin signaling, cell adhesion, proliferation, survival and migration [17–19]. FAK homologs share approximately 95~97% sequence identity across different organisms [20]. It has been reported that overexpression of FAK is associated with several types of tumors and is implicated in tumorgenesis and metastasis [21]. Inhibiting FAK function, either by small molecular inhibitor, targeting FAK *via* RNAi or expressing dominant negative FRNK, reduced tumor progression and metastasis.

MicroRNAs (miRNAs) are a class of small, endogenous, non-coding RNAs which typically down-regulate the expression of their target genes at the post-transcriptional level. Most of them have a region made of 2~8 nucleotides called “seed” region binding to completely or partially complementary regions in the 3′ untranslated region (UTR) of those target genes [22]. In the past a few years, miRNAs have been verified to play essential roles in a variety of cellular and pathological processes, such as tumor progression and metastasis [23, 24]. The mechanism of miRNA regulation is still a relative new and rapidly growing research area far from complete elucidation.

According to an online cancer transcriptome database Oncomine, FADD and FAK are both over-expressed in human melanoma. In this paper, we report that FAK was down-regulated in FADD-deficient MEF cells (FADD^−/−^ MEFs). Microarray analysis revealed an up-regulation of miR-7a expression in FADD^−/−^ MEFs. FADD deficiency inhibited FAK expression by promoting miR-7a in two murine melanoma cells with the same origin and genetic background but different metastatic potency, B16F10 and B16F1. Interestingly, we also observed suppression of FAK expression which retarded cell migration caused by FADD interference can be abrogated by recovering miR-7a expression level. We suggest that FADD may play a novel role in cell migration by regulating FAK expression at which miR-7a acts as a mediator.

## RESULTS

### FADD and FAK overexpression was a novel prognostic factor in several types of cancers including melanoma

**Figure 1:**
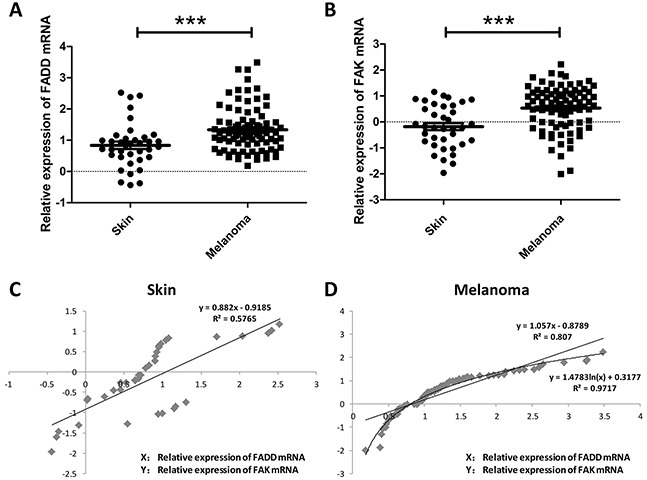
FADD and FAK overexpression was associated with human melanoma cancer progression **A.** Oncomine analysis of FADD expression in human Melanoma cancer. **B.** Oncomine analysis of FAK expression in human Melanoma cancer. **C.** Correlation analysis of FADD and FAK expression in human normal skin. **D.** Correlation analysis of FADD and FAK expression in human melanoma cancer.

FADD overexpression has been observed in head and neck squamous cell carcinoma, breast cancer, lung cancer and early-stage glottic squamous cell carcinoma and correlates with poor survival rate [12, 14–16, 25, 26]. It was reported that high levels of Fas/DR5/FADD/caspase-8 death signaling play a critical role in regulation of cancer metastasis in human head and neck cancer [15]. And it has been unraveled for years that FAK signaling pathway is a frequently altered pathway in tumor metastasis and invasion in various types of tumors, with the overexpression of FAK in the tumor tissues and lymph nodes. Here we raised the question regarding the correlation between expression of FADD and FAK in melanoma. To check the FADD expression in human melanoma, we first performed analysis of published patients' data using Oncomine (http://www.oncomine.org), a free online bioinformatic resource of cancer transcriptome data. It collects clinical mRNA array data of different genes from different patients all over the world. After registering an account on Oncomine, researchers may view a variety of results and quickly identify studies or analysis of interest. Researchers could also share their own results and findings by uploading data to Oncomine. As shown in Figure 1A, FADD mRNA level was significantly elevated in human melanoma cancer (p<0.001). Increased FAK expression was also evident in human melanoma compared to normal skin cells according to Oncomine (Figure 1B, p<0.0001). Figure 1C and Figure 1D showed the correlation of the two genes in both skin and melanoma conditions. And it appears that correlation between these two genes is more evident in melanoma cells. Taken together, these findings indicated a functional linkage between FADD overexpression and melanoma cancer progression which in turn suggested a positive correlation between FADD and FAK expression.

### FADD-deficiency decreased FAK mRNA and protein levels in murine embryonic fibroblast cells

**Figure 2:**
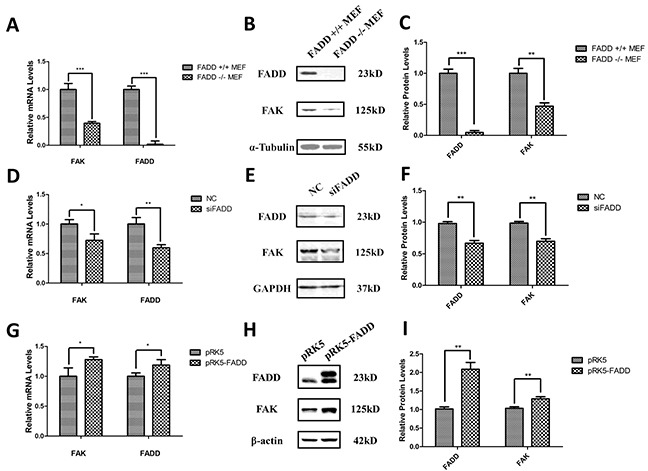
FADD regulated FAK expression in MEF cells **A.** mRNA level of FAK down-regulated in FADD^−/−^ MEF cells. **B.** and **C.** Representative western blot analysis (B) and quantification of expression (C) of FADD and FAK in FADD^+/+^ and ^−/−^ MEF cells. α-Tubulin was used as a loading control and the FADD^+/+^ MEF cell served as the sample control. **D.** FADD was interfered by 20 μM siRNA (siFADD) in MEF cells for 48 h, scramble siRNAs served as negative control (NC). The mRNA expression of FAK decreased in siFADD samples compared with NC. **E.** and **F.** Representative western blot analysis (E) and quantification of expression (F) of FADD and FAK in FADD knockdown MEF cells by siRNA. GAPDH used as a loading control. MEFs treated with scramble siRNAs served as the sample control. **G.** The cloned pRK5-FADD plasmid was transfected into MEFs to make FADD overexpressed and pRK5 empty vector plasmid as negative control. The mRNA level of FAK increased in cells with pRK5-FADD plasmid compared to the cells with empty plasmid. **H.** and **I.** Representative western blot analysis and quantification of expression of FADD and FAK in FADD overexpression MEF cells. GAPDH used as a loading control. MEFs transfected with pRK5 empty vector plasmid acted as the sample control. mRNA levels were monitored by qRT-PCR and proteins were detected with indicated antibodies by western blotting. Band intensity was quantified by Image J software. The results shown are representative of three different experiments. Data are represented as mean ± S.D. *p < 0.05; **p < 0.01, ***p < 0.001.

To study the correlation between FADD and FAK expression, FADD-deficient MEF cells (FADD^−/−^ MEFs) were used. Interestingly, we found that both mRNA and protein levels of FAK were decreased in FADD^−/−^ MEFs compared with FADD^+/+^ MEFs (Figure 2A, 2B and 2C). Furthermore, we used siRNA of FADD (siFADD) to silence FADD expression in MEFs and monitored the expression of FAK. Similarly, the mRNA and protein levels of FAK were also down-regulated in MEFs transfected with FADD siRNAs (Figure 2D, 2E and 2F). In contrast, FAK expression was increased in MEF cells with FADD overexpression. We amplified the sequence of murine FADD from the cDNA of B16F10 cells and cloned into pRK5 empty vector plasmid, constructing the pRK5-FADD plasmid. pRK5 and pRK5-FADD plasmids were then transfected into normal MEF cells to elucidate the correlation between FAK and FADD expression. As shown in Figure 2G, the mRNA and protein levels of FAK increased in MEFs transfected with pRK5-FADD plasmids (Figure 2H and 2I).

### Expression of miR-7a was up-regulated in FADD-deficient MEFs

To identify miRNAs potentially involved in the FADD/FAK network, we performed miRNA microarray profiling in FADD^+/+^ MEF and FADD^−/−^ MEF cells. A total of four miRNAs were differentially expressed (fold change of ≥ 2, p < 0.01) in FADD-deficient MEF cells, including down-regulation of three miRNAs and up-regulation of one miRNA (Table 1).

**Table 1:** Differential miRNA expression in murine fibroblast cell line FADD^+/+^ MEFs versus FADD^−/−^ MEFs

No.	Probe_ID	FADD^−/−^ MEF Signal	FADD^+/+^ MEF Signal	Log_2_[(FADD^+/+^ MEF)/(FADD^−/−^ MEF)]
**1**	mmu-let-7b	808.60	18231.82	4.54
**2**	mmu-let-7c	3489.63	21282.04	2.58
**3**	mmu-miR-7a	2541.13	432.78	−2.58
**4**	mmu-miR-19b	71.51	342.66	2.26

**Figure 3:**
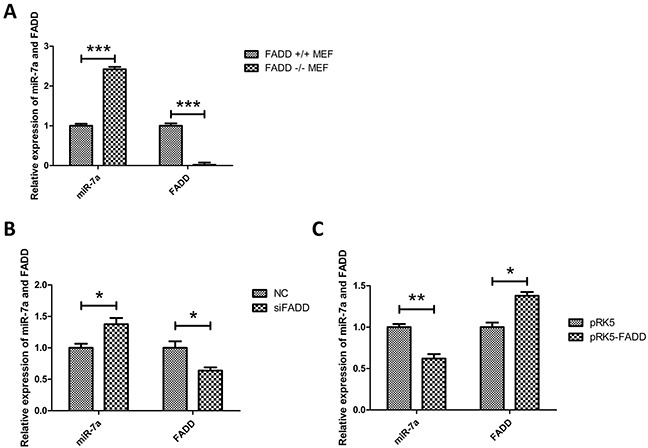
Expression of miR-7a was up-regulated in FADD−/− MEFs **A.** Validation of expression of the only up-regulated miRNA, miR-7a, screened by microarray analysis in FADD^−/−^ MEFs compared with FADD^+/+^ MEFs (Table 1) detected by qRT-PCR. **B.** and **C.** When FADD was knockdown by RNAi (B) or overexpressed by transfected pRK5-FADD plasmid (C), the expression of miR-7a reversely correlated to FADD expression in MEFs. The results shown are representative of three different experiments. Data are represented as mean ± S.D. *p < 0.05; **p < 0.01, ***p < 0.001.

To confirm microarray results, qRT-PCR experiments were performed as shown in Figure 3A. The results from FADD-knockout cells only can illustrate the expression of these four miRNAs under the condition of the absence of FADD. To further understand the correlation between the expressions of the four miRNAs mentioned above with the FADD expression level, we took the other two situations, FADD-knockdown or FADD low-expression and FADD-overexpression, into consideration. The results of miR-7a expression detected by qRT-PCR under these two conditions were shown in the Figure 3B and 3C, unraveling a negative relationship between miR-7a and FADD expression levels in MEF cells.

Given that Keith M. Giles *et al*. [27] have identified miR-7 as a tumor suppressor miRNA which inhibits human melanoma cell migration and invasion, and considering the results mentioned above, we selected miR-7a for further analysis.

### The relationship between FADD, FAK and miR-7a in murine melanoma cells

**Figure 4:**
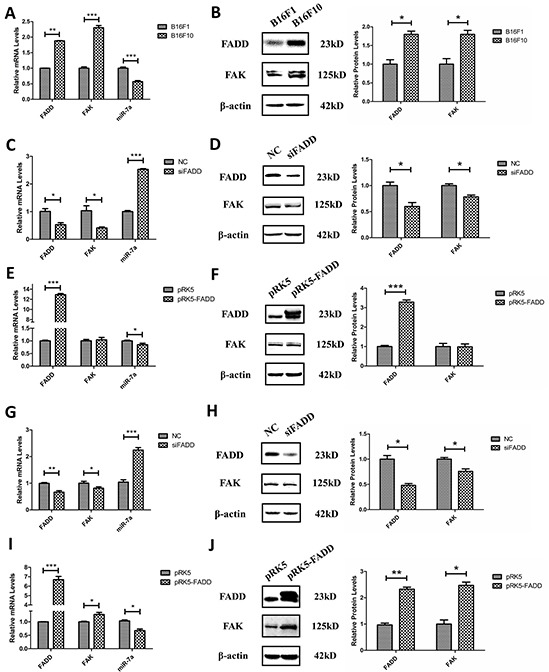
FADD regulated FAK and miR-7a expression in B16F10 and B16F1 melanoma cells **A.** and **B.** In highly metastatic B16F10 cells, FADD and FAK exhibited higher level of mRNA (A) and protein (B) expression compared to lowly metastatic B16F1 cells. Conversely, the expression of miR-7a is lower in B16F10 cells than in B16F1. B16F1 cells served as the control sample. **C.** FADD was interfered by 20 mM siRNA (siFADD) in B16F10 cells for 48 h, scramble siRNAs as negative control (NC). The mRNA expression of FAK decreased in cells with siFADD compared to NC group while the expression of miR-7a increased. **D.** Representative western blot analysis and quantification of expression of FADD and FAK in FADD knockdown B16F10 cells by siRNA. β-actin used as a loading control. **E.** FADD was overexpressed by transfected pRK5- FADD plasmid into B16F10 cells for 48 h. The mRNA expression of FAK increased in cells with pRK5-FADD plasmid compared to those with pRK5 empty vector plasmid while miR-7a decreased. **F.** Representative western blot analysis and quantification of expression of FADD and FAK in FADD overexpressed B16F10 cells. β-actin acted as a loading control. **G.** and **I.** The mRNA level of FAK expression also increased while FADD up-regulated (I) and decreased while FADD down-regulated (G) in B16F1 cells. **H.** and **J.** Representative western blot analysis and quantification of expression of FADD and FAK in FADD knockdown (H) or overexpressed (J) B16F1 cells. β-actin served as a loading control. Cells treated with siRNA scramble negative control (NC) and pRK5 empty vector plasmid served as the sample control. mRNA levels were detected by qRT-PCR and proteins were detected with indicated antibodies by western blotting. Band intensity was quantified by Image J software. The results shown are representative of three different experiments. Data are represented as mean ± S.D. *p < 0.05; **p < 0.01, ***p < 0.001.

As previously mentioned, we demonstrated that expressions of FAK and FADD were positively correlated while the expression of miR-7a exhibited a negative correlation with these two proteins in MEFs. Here we focused on two murine melanoma cells, highly metastatic B16F10 and lowly metastatic B16F1, attempting to elucidate their expression profiles and connections. Firstly, we measured the basic expression of FADD, FAK and miR-7a in these two murine melanoma cells. As expected, FADD and FAK showed higher expression level in B16F10 cells than in B16F1 cells, confirmed by both mRNA and protein expression measurements (Figure 4A and 4B). Expression of miR-7a, on the contrary, was reduced in B16F10 cells compared to B16F1 cells (Figure 4A). This result was consistent with the metastatic capability of B16F10 and B16F1 cells. Secondly, knockdown and overexpression of FADD were both performed in B16F10s and B16F1 cells, using siRNAs (NC and siFADD) and plasmids (pRK5 empty vector plasmids and pRK5-FADD plasmids). As shown from Figure 4C to Figure 4J, the qRT-PCR and western blotting assay indicated a positive correlation between FAK and FADD expression but a negative correlation between miR-7a and FADD in these two cells. FADD knockdown was associated with down-regulation of FAK but up-regulation of miR-7a (Figure 4C, 4D, 4G and 4H) while FADD overexpression was associated with up-regulation of FAK but down-regulation of miR-7a (Figure 4E, 4F, 4I and 4J), consistent with the results from MEF cells.

### FAK was a target gene of miR-7a in B16F10 cells

Several groups have reported that miR-7 inhibits migration and invasion *via* targeting FAK expression in human breast, cervical and glioblastoma cancer cells [28–30]. Therefore, we assumed a similar role of miR-7a in murine melanoma cells. B16F10 was used due to its high expression profile of FAK. We employed programs miRanda and PicTar to predict target sites of miR-7a on FAK mRNA.

**Figure 5:**
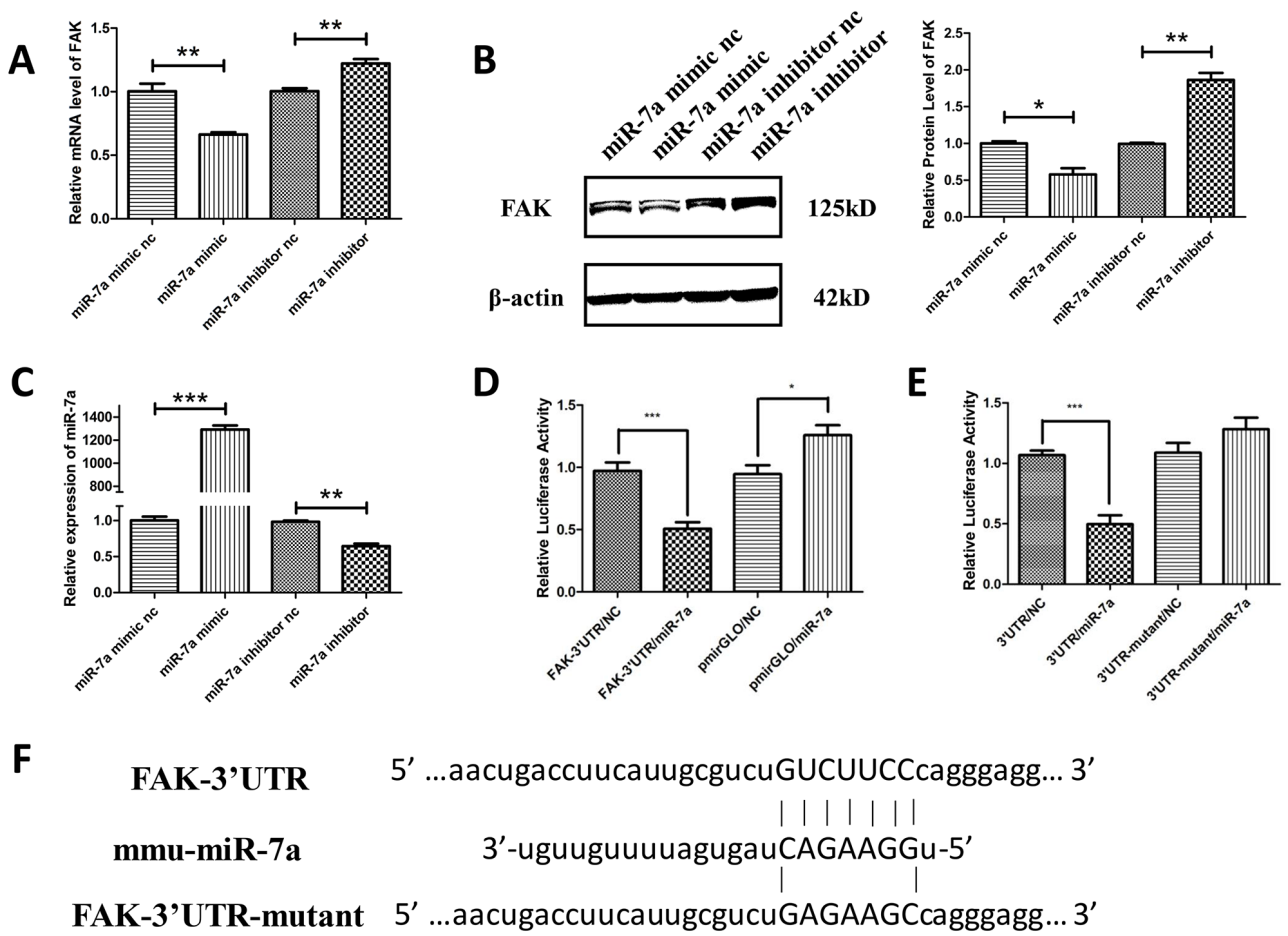
FAK was the target gene of miR-7a in murine melanoma B16F10 cells **A.** and **B.** qRT-PCR and western blotting analyses of FAK expression in B16F10 cells treated with miR-7a mimic and inhibitor. FAK protein and mRNA levels were reduced in miR-7a mimic-transfected cells while upregulated in miR-7a inhibitor-transfected cells compared to each negative controls. **C.** The expression level of miR-7a after treated by miR-7a mimic and inhibitor detected by qRT-PCR. **D.** Luciferase reporter assay of co-transfected FAK-3′UTR plasmid with miR-7a mimic or mimic nc, pmiRGLO together with miR-7a mimic or nc as the negative control. **E.** Luciferase reporter assay of co-transfected FAK-3′UTR or FAK-3′UTR-mutant plasmid with miR-7a mimic or miR-7a mimic nc. The luciferase activity was significantly decreased in co-transfected with FAK-3′-UTR and miR-7a mimic F10 cells compared to the negative control miRNA. No such changes were noted for the mutant 3′-UTR of FAK and pmiRGLO vector. **F.** Alignments of nucleotides 749-755 in the FAK-3′UTR with mmu-miR-7a, and the mutation in the miR-7a-binding site of FAK-3′UTR-mut. mRNA levels were detected by qRT-PCR and proteins were detected with indicated antibodies by western blotting. Band intensity was quantified by Image J software. The results shown are representative of three different experiments. Data are represented as mean ± S.D. *p < 0.05; **p < 0.01, ***p < 0.001.

A potential miR-7a target site was found at position 749-755 of the FAK 3′-UTR. To validate whether FAK is a miR-7a target gene in murine melanoma cells, FAK mRNA and protein expression was determined in B16F10 cells after knockdown or overexpression of miR-7a. As shown in Figure 5A, miR-7a mimic significantly reduced the mRNA level of FAK. Conversely, FAK protein expression was up-regulated in B16F10 cells transfected with miR-7a inhibitor. Likewise, protein level of FAK was in accordance with its mRNA level determined by western blot (Figure 5B). Figure 5C shows the expression of miR-7a detected by qRT-PCR in B16F10 cells treated with miR-7a mimic and inhibitor. To verify whether the predicted miR-7a binding sites in the 3′-UTR of FAK were responsible for the regulation, we cloned the original sequence of the predicted miR-7a binding site as well as a mutated version into a luciferase reporter vector (FAK-3′UTR and FAK-3′UTR-mutant) (Figure 5F). The luciferase activity was significantly decreased in B16F10 cells co-transfected with FAK-3′-UTR and miR-7a mimic compared to the negative control miRNA. No change in luciferase activity was observed for the mutant 3′-UTR of FAK (3′UTR-mutant) and vector control (pMIRGLO) (Figure 5D and 5E). Taken together, these data indicated that FAK is a miR-7a target in B16F10 murine melanoma cells.

### FADD promoted cell migration in murine melanoma cells while miR-7a inhibited migration

**Figure 6:**
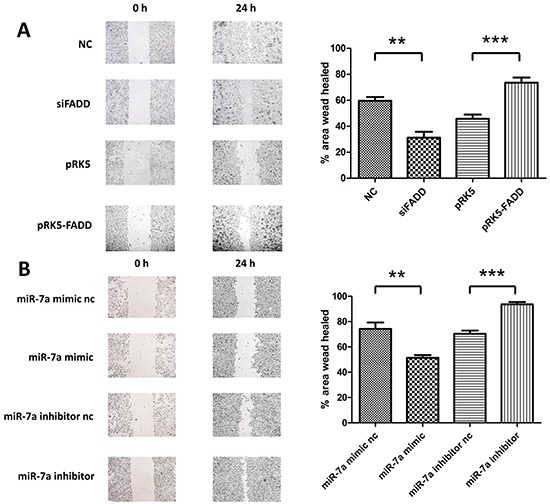
FADD promoted cell migration in murine melanoma cells while miR-7a inhibited it **A.** Migration analysis of B16F10 and B16F1 by wound healing assay at 24 hours. B16F10s which were transfected with FADD siRNA (siFADD) showed lower migrating potential compared to the negative control scramble siRNA (NC) while the migration rate of those B16F1 cells transfected with pRK5-FADD was faster than the transfected with pRK5 empty vector plasmid ones. **B.** Migration analysis of B16F10 transfected with miR-7a mimic or inhibitor by wound healing assay at 24 hours. Cells overexpressed miR-7a migrated slower than its negative control while miR-7a knockdown provided a faster rate of migration compared to the miR-7a inhibitor negative control. Band intensity was quantified by Image J software. The results shown are representative of three independent experiments. Data are represented as mean ± S.D. *p < 0.05; **p < 0.01, ***p < 0.001.

The above studies have shown that FADD regulates mRNA and protein expression levels of FAK in murine melanoma cells. Given the oncogenic nature of FAK, we investigated whether FADD influences cell migration. B16F1 and B16F10 cells transfected with the overexpression pRK5-FADD plasmid or FADD siRNA. As expected, opposite effects were observed for FADD overexpression and knockdown (Figure 6A). Mueller, D.W. *et al.* [2] demonstrated that miR-7 expression was decreased in human melanoma cells and associated with tumor metastasis. Keith M. Giles. *et al*. [27] further announced miR-7 had a significant down-regulation in two human metastatic cell lines compared with the other primary melanoma-derived cell lines. Here we also confirmed the anti-migratory function of miR-7a in murine melanoma cells. We increased miR-7a expression in murine B16F10 cells using miR-7a mimic and decreased it with miR-7a inhibitor. After a 24 h period, we found that miR-7a overexpression substantially reduced the rate of cell migration, while miR-7a knockdown increased the rate of migration (Figure 6B).

### MiR-7a was a mediator in FADD-regulated expression of FAK

**Figure 7:**
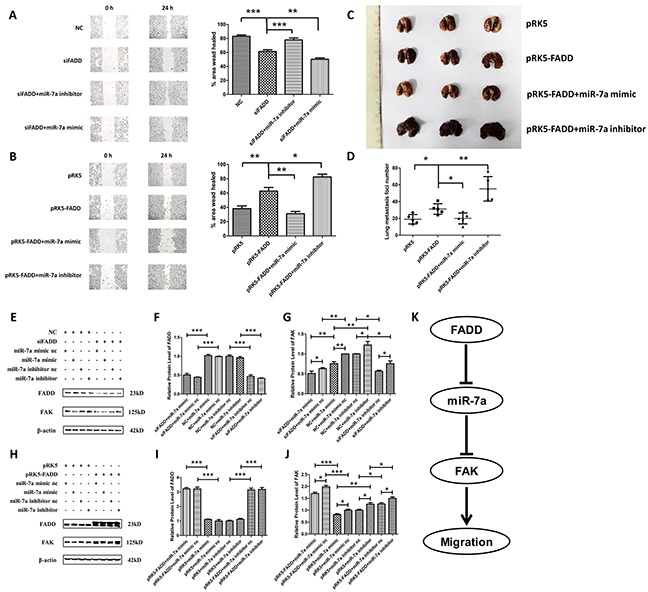
MiR-7a was a mediator in FADD-regulated expression of FAK **A.** Firstly, transfected FADD siRNA (siFADD) alone or co-transfected with FADD siRNA (siFADD) and miR-7a mimic or inhibitor into B16F10 cells for 48 hours. Then a 24 h period scratch wound healing assay had been launched in those transfected B16F10 cells after the transfection. Inhibiting miR-7a expression rescued FADD interference reduced cell migration. Migration was retarded even more when miR-7a mimic were used instead of inhibitor co-transfected with FADD siRNAs. **B.** Firstly transfected pRK5-FADD plasmid alone or co-transfected pRK5-FADD plasmid together with miR-7a mimic or inhibitor into B16F1 cells for 48 hours. Then a 24 h period scratch wound healing assay had been launched in those B16F1 cells after the transfection. FADD overexpression promoted cell migration which is repressible by miR-7a mimic. Cells co-transfected with pRK5-FADD and miR-7a inhibitor exhibited the fastest migration rate among the four different samples. **C.** and **D.** B16F1 melanoma cells treated with pRK5 empty vector plasmids, pRK5-FADD plasmids, pRK5-FADD plasmids plus miR-7a mimic and pRK5-FADD plasmids plus miR-7a inhibitor, were collected and suspended in PBS. Then the treated cells were injected into the tail vein of C57BL/6 mice. After 14 days, mice were sacrificed with all the lung tissues were excised and the surface lung tumor nodules were counted. The representative pictures of lung metastases (C) and the number of metastasis foci on the surface of lung (D). Data are presented as mean ± S.D. n=5. **E, F.** and **G.** Representative western blot analysis (E) and quantification of expression of FADD (F) and FAK (G) in the miR-7a inhibitor/mimic co-transfected with FADD siRNAs experiment in B16F10s assessed by western blot. **H, I.** and **J.** Representative western blot analysis (H) and quantification of expression of FADD (I) and FAK (J) in the miR-7a inhibitor or mimic co-transfected with pRK5-FADD experiment in B16F1s assessed by western blot. β-actin served as a loading control. Cells treated with siFADD scramble siRNA negative control (NC) and miR-7a mimic or inhibitor nc served as the sample control in the former experiment, while those treated with pRK5 empty vector plasmid and miR-7a mimic or inhibitor nc served as the sample control in the latter one. **K.** The systematic schema of the relations between FADD, FAK and miR-7a. Band intensity was quantified by Image J software. The results shown are representative of three different experiments. Data are represented as mean ± S.D. *p < 0.05; **p < 0.01, ***p < 0.001.

A strong increase of miR-7a expression in FADD-deficient B16F1 and B16F10 cells was observed, as reported above. We then pursued an investigation on whether miR-7a is essential in the FADD-mediated regulation of cell migration *via* FAK. Since B16F10 was highly metastatic with higher FAK expression than B16F1, we co-transfected FADD siRNA (siFADD) with either miR-7a mimic or inhibitor into B16F10 cells. On the other hand, pRK5-FADD plasmid was co-transfected with either miR-7a mimic or inhibitor into B16F1 cells. Wound healing assay in serum-free DMEM was performed 48 h after transfection. Scramble siRNA and empty vector plasmid (pRK5) were used as negative controls. According to our hypothesis, if the weakening of FAK-induced migration by FADD inhibition was mediated by miR-7a, miR-7a inhibitor would rescue cells from retarded migration whereas miR-7a mimic would further inhibit the migratory ability. Similarly, up- or down-regulated miR-7a expression with mimic or inhibitor would have the homoplastic effect on FADD-overexpressed cells. As shown in Figure 7A, after a 24 h period, cells treated with siFADD migrated significantly slower than siRNA NC, and the migration of those treated with siFADD and miR-7a inhibitor was faster than siFADD. siFADD plus miR-7a mimic conferred even more potent migratory inhibition than siFADD alone. In contrast, cells co-transfected with FADD plasmid (pRK5-FADD) plus miR-7a inhibitor migrated remarkably faster than those transfected with FADD plasmids alone. FADD overexpression increased the migratory ability of B16F1 cells which can be inhibited by miR-7a mimic (Figure 7B). To further verify our results, we created a mice lung metastatic model *in vivo*. Twenty female C57BL/6 mice were divided into 4 groups (5 animals/group). We injected the four groups of mice with B16F1 melanoma cells transfected with pRK5 empty vector plasmids, pRK5-FADD plasmids, pRK5-FADD plasmids plus miR-7a mimic, or pRK5-FADD plasmids plus miR-7a inhibitor through tail vein. After 14 days, all mice were sacrificed and lung tissues were excised. Surface lung tumor nodules were counted to quantify metastatic ability at which more tumor nodules correspond to higher metastatic ability. As shown in Figure 7C and 7D, *in vivo* result was consistent with that *in intro* (Figure 7B). Mice injected with pRK5-FADD-transfected cells had more tumor nodules than those in the pRK5 group, whereas the number of tumor nodules seemed to be reduced by miR-7a mimic but increased by miR-7a inhibitor. Western blotting analysis was used to demonstrate the protein levels of FADD and FAK in these two experiments (Figure 7E and Figure 7H). According to the quantitative analysis, protein level of FAK in these two experiments was consistent with their respective metastatic ability. As shown in Figure 7G, B16F10 cells treated with FADD siRNA scramble (NC) plus miR-7a inhibitor exhibited higher protein level of FAK than NC plus miR-7a inhibitor nc, but the protein level of FAK in B16F10 cells treated with siFADD plus miR-7a inhibitor significantly decreased, though it did not decrease as much as that in B16F10 cells treated with its control DNA (siFADD plus miR-7a inhibitor nc). We then monitored FAK expression of cells co-transfected with miR-7a mimic and FADD siRNA. It was not surprising that FAK expression was dramatically reduced compared to the cells treated with NC and miR-7a mimic, where the latter ones already exhibited decreased FAK expression compared to the two basic NC and miR-7a mimic nc. Likewise, Figure 7J showed that the up or down-regulation of miR-7a could decrease or increase the expression of FADD-induced FAK in B16F1 cells, respectively. To sum up, these results suggest that miR-7a was an important mediator in FADD-regulated expression of FAK in both B16F10 and B16F1 cells. The FADD/miR-7a/FAK pathway acted as a regulatory pathway of migration in murine melanoma cells, and this finding was consolidated by an *in vivo* mouse metastatic model.

## DISCUSSION

FADD is originally identified as an adaptor protein in the apoptotic signaling pathway which couples Fas receptor and caspase-8 death signals. Its essentiality in apoptosis has been well-documented [6, 31, 32]. Interestingly, FADD has also been reported to play an emerging role in non-apoptotic processes such as cell cycle, embryogenesis, lymphocyte development and proliferation [10, 33, 34]. FADD overexpression has been observed in head and neck squamous cell carcinoma, breast cancer, lung cancer and early-stage glottic squamous cell carcinoma and is associated with poor survival [12, 14–16, 25, 26]. It has been reported that Fas/DR5/FADD/caspase-8 death signaling pathway plays a critical role in regulation of cancer metastasis in human head and neck cancer [15]. Pattje WJ, *et al.* [16] and Zhang, C, *et al.* [35] also demonstrated that FADD was associated with a higher incidence of lymph node metastases and might cause an increased risk of distant metastasis in head and neck squamous cell carcinoma. And Fas/FasL complex promoted proliferation and migration *via* the FADD/FLIP/TRAF/NF-kB pathway in brain endothelial cells. However, the mechanisms between FADD and tumor metastasis or cell migration are still unclear, especially in melanoma. This study illustrates a potential role of FADD in murine melanoma cell migration by regulating FAK expression; a pathway that involves miR-7a as a crucial mediator.

FAK signaling pathway was frequently reported to promote cell migration or tumor metastasis and invasion in various types of tumors, with FAK overexpressed in the tumor tissues and lymph nodes. In this report, we discovered that the expression of FAK, which was closely related to cell spreading and migration, was down-regulated in FADD-deficient murine fibroblast cell line (FADD^−/−^ MEFs) (Figure 2A). Subsequently, we investigated both mRNA and protein levels of FAK in MEFs depleted of FADD or bearing FADD overexpression and the results indicated that FAK expression was correlated with FADD positively. Based on these results and the numerous reports on FAK's importance in and contribution to cell adhesion, spreading, proliferation, migration and invasion, we began to explore the mechanism of FADD's involvement in cell migration through regulation of FAK expression. We also investigated the relationship between FADD and FAK expression in two murine melanoma cell lines, B16F10 and B16F1, and obtained consistent results with MEFs. Although the up-regulation of FAK in B16F10 caused by extrinsic transfection of pRK5-FADD plasmid was not obvious compared to the empty vector plasmid (pRK5) transfection, it does not contradict our hypothesis, to some extent. We have to admit that the difference between the highly metastatic melanoma cell line B16F10 and the lowly metastatic cell line B16F1 may exist, though they are both obtained from B16F0. The highly metastatic melanoma cell line B16F10 expresses high basic levels of FADD and FAK, low basic levels of miR-7a. The decrease in miR-7a caused by artificial overexpression of FADD in B16F10 was not obvious compared to that in lowly metastatic cell line B16F1, leading to an inconspicuous change in FAK expression in B16F10 (Figure 4E and 4F), compared to a profound change in FAK in B16F1 (Figure 4I and 4J). However, when miR-7a was directly inhibited by an inhibitor a much more prominent change in FAK can be observed in B16F10 (Figure 5B). Therefore, it is reasonable to conclude that the miR-7a's response to FADD overexpression in B16F10 cells was not as sensitive as that in B16F1 cells. But when FADD expression was suppressed in these two cells, miR-7a's responses were sensitive in both B16F10 and B16F1 cells (shown in Figure 4C, 4D, 4G and 4H). As a result, FAK expression decreased in both these two melanoma cells. Detailed mechanism involving FADD/miR-7a/FAK in these two melanoma cells still remains exclusive and requires further investigation.

With the help of microarray and qRT-PCR analysis, we predicted that miR-7a, a reported tumor suppressor miRNA which inhibits tumor migration and invasion, might be an indispensable player in the interplay between FADD and FAK (Table 1 and Figure 3A, 3B and 3C). Hsa-miR-7 (the same sequence as mmu-miR-7a) has been reported to inhibit EMT transition, metastasis and invasion in human breast, cervical cancer and glioblastoma cells *via* targeting focal adhesion kinase [28, 29, 36]. Both miR-7 and FAK have been reported to regulate melanoma migration and invasion, but the effects exerted by miR-7 and FAK in melanoma are opposite. miR-7 prohibits migration while FAK promotes it [27, 36–38]. We reasoned that miR-7a must bear a similar function by targeting FAK expression in melanoma as it does in other types of cancer. To verify our assumption, we transfected the mimic and inhibitor of miR-7a into B16F10 cells followed by analysis of mRNA and protein levels of FAK (Figure 5A and 5B). The results confirmed our assumption. We then cloned FAK-3′UTR and FAK-3′UTR-mutant into luciferase reporter plasmids, co-transfected them with miR-7a mimic and investigated their luciferase activities. The decrease of luciferase activities in cells co-transfected with functional FAK-3′ UTR plasmid and miR-7a mimic plasmid confirmed FAK is the target gene of miR-7a in melanoma as well.

Since both miR-7a and FAK were reported to be involved in cell migration in melanoma [27, 36, 37, 39], and our previous study demonstrated FADD's correlations with the two of them. Therefore it was reasonable to assume that FADD could have impact on cell migration *via* miR-7a targeting FAK signaling pathway in murine melanoma cells. We used wound healing assay to probe cell migratory ability under FADD and miR-7a knockdown or overexpression conditions. Our results revealed that the migration rate of B16F10 cells was significantly reduced when FADD expression was interfered by RNAi, but the migration rate was restored when cells were co-transfected with FADD siRNA and miR-7a inhibitor (Figure 7A). FAK expression correlates positively with cell migratory ability (Figure 7E, 7F and 7G). Since the basic FAK expression level in B16F10 was originally high, we chose B16F1 to study FAK expression and cell migratory ability in cells with up and down-regulated miR-7a along with FADD overexpression (Figure 7B, 7H, 7I and 7J). To further verify our *in vitro* results, we conducted a mice lung metastasis model experiment. The results from animal experiment (Figure 7C and 7D) appeared to be consistent with the cell migration results *in vitro* very well (Figure 7B). To summarize, both *in vitro* and *in vivo* data suggest a crucial role of miR-7a in FADD-regulated expression of FAK and cell migration.

In conclusion, we provide a new model of regulation of cell migration by the FADD-miR-7a-FAK pathway in murine melanoma cells. As the systematic schema showed in Figure 7K, by elevating the expression of miR-7a, FADD interference could decrease the expression of FAK expression. Although we have not elucidated a detailed mechanism of how FADD regulates the expression of miR-7a, we suspect that FADD may regulate miR-7a's expression by altering transcriptional activity of hnRNP K pre-mRNA transcript from which miR-7a is derived [40]. We have reasons to believe that miR-7a could be regulated by FADD at the transcription level and further efforts are required to unravel a more comprehensive mechanism. As for the relationship between miR-7a and FAK, miR-7a could suppress FAK expression through binding to 3′UTR of FAK mRNA and lead to subsequent mRNA degradation. Down-regulated FAK further inhibits cell migration. This model also helps to explain why FADD interference may reduce the metastasis of melanomatosis and opens new perspectives for cancer therapy. Although murine melanoma cells lines were used in this work, studies using human melanoma cells lines (e.g. A375, A2058 and Lu1205) have reported similar results, where cell migration can be inhibited by FAK suppression in combination with some other miRNA or small molecule [41–43]. Furthermore, several groups have used both B16F10 and A375 cell lines to study the molecular mechanisms of melanoma cells, assuring a great experimental value of murine melanoma cells [44–46]. Therefore, we believe our study in murine melanoma is of significant clinical meaning. Importantly, our work suggests that the FADD-miR-7a-FAK pathway can be a promising therapeutic target for melanoma cancer therapy.

## MATERIALS AND METHODS

### Cell lines and animals

Murine fibroblast cell line MEF and melanoma cell line B16F10, B16F1 were purchased from the American Type Culture Collection. Construction and validation of the MEF cell lines were performed in Dr. Astar Winoto's Laboratory (UC Berkeley, Berkeley, CA) as previously described [47]. Cells were stored and recovered from cryopreservation in liquid nitrogen and cultured in DMEM (Wisent, Canada) medium plus 10% FBS (Invitrogen, USA), 50 mg/ml streptomycin and 50 U/ml penicillin and cultured in 5% CO_2_ humidified atmosphere. Six-week-old healthy female C57/B6 mice were purchased from Vitalriver (Beijing, China) and maintained in pathogen-free conditions for one week prior to experiments. All animal procedures were performed according to the protocol approved by the Nanjing University Animal Care and Use Committee.

### miRNA microarray analysis

FADD^+/+^ MEF and FADD^−/−^ MEF cells were seeded in two 60 mm dishes (5 × 10^6^ each) for 24 h before collection. After 24 h, cells were washed twice with PBS and lysed with 1 ml Trizol for 15 min and harvested. And the miRNA microarray analysis was carried out in Shanghai Kangcheng Co. Ltd.

### Plasmid construction and luciferase reporter assay

The sequence of murine FADD was amplified from cDNA of B16F10 cells and cloned into pRK5 empty vector. The postulated miR-7a target sequence of the murine FAK 3′ untranslated region (3′UTR) was 5′-GUCUUCC-3′, corresponding to nucleotides 749-755, as predicted by miRanda and PicTar. To show that miR-7a inhibits the expression of the murine FAK gene by directly binding to its 3′UTR, the murine FAK 3′UTR was amplified and cloned into the pmirGLO Vector (Promega, USA), downstream of the luciferase gene, named pGL-FAK-3′UTR. And another vector named pGL-FAK-3′UTR-mutant was generated from pGL-FAK-3′UTR by deleting the putative binding site for miR-7a “GUCUUCC”. The primers used were as follows: FADD, 5′-AATTGAGCTCATGGACCCATTCCTGGTGCT-3′ (Forward); 5′-GCTTAAGCTTTCAGGGTGTTTCTGAGGAAGAC-3 (Reverse); FAK-3′UTR, 5′-CGAGCTCGCCCCTGGCCATTGAACG-3′ (Forward); 5′-CCTCGAGCCCGGCGCCACCTTTTTA-3′ (Reverse); FAK-3′UTR-mutant, 5′-GTCTCAGAAGTCAGGGAGGACCCCGCAGGACAA-3′ (Forward); 5′-CCTGCCTTCTAAGACGCAATGAAGGTCAGTTAG-3′ (Reverse).

For the luciferase reporter assay, cells were originally seeded in a 24-well plate for 24 h. In each well, luciferase reporter vector and miR-7a mimic were co-transfected using Lipofectamine 2000 reagent (Invitrogen, USA). 48 h post-transfection, B16F10 cells were washed twice with PBS and lysed with 100 mL Passive Lysis Buffer for 15 min and harvested. Firefly and Renillaluciferase activities were measured using the GloMaxTM 20/20 Dual-Luciferase Reporter assay system (Promega) according to the manufacturer's protocols. Firefly luciferase activity was normalized to Renillaluciferase activity.

### Transfections of miRNA, small interfering RNA (siRNA) and murine FADD expression plasmid

All synthetic miRNA products including miR-7a mimic (dsRNA oligonucleotides), negative control mimic (NC) (micrON™ mimic Negative Control #22, Standard, 2nmol), miR-7a inhibitors, inhibitor negative control (micrOFF™ inhibitor Negative Control #22, Standard, 2nmol) were purchased from RiBoBio (Guangzhou, China). siRNAs and the negative control (NC) were purchased from Shanghai GenePharma Co. Ltd. The sequences of siRNA and miRNAs are as follows: FADD: 5′-ACGAUCUGAUGGAGCUCAA-3′; miR-7a (MIMAT0000677): 5′-UGGAAGACUAGUGAU UUUGUUGU-3′. Cells were seeded into six- or twelve-well plates the day before transfection, ensuring about 50% cell confluence at the moment of transfection. For transfection, cells were transiently transfected with miRNAs, siRNAs or plasmids using lipofectamine 2000 (Invitrogen, USA) according to the manufacturer's instructions. The oligonucleotides were used at a final concentration of 50 nM.

### RNA isolation and quantitative real-time PCR

Total RNA was isolated with Trizol reagent (Invitrogen, USA) following the manufacturer's instruction. Quantitative real-time PCR was performed using reverse transcription kit (Takara, Japan) and SYBR Green PCR Master Mix (Roche, Germany). For quantitation of mRNA, 1 mg total RNA was reverse-transcribed with random primers while the stem-loop method was used for miRNA. The PCR cycling conditions were as following, an initial hot start at 95°C for 10 min, followed by 40 cycles at 95°C for 10 s and 60°C for 30 s. Quantification was done using the 2^−ΔΔCt^ relative quantification method with mouse β-actin as an internal control for mRNAs while U6 as an internal control for miRNAs. The primer sequences are as follows: FADD, 5′-CGGGCAACGATCTGATGGA-3′ (forward) and 5′-ACAATGTCAAATGCCACCTGC-3′ (reverse); FAK, 5′-CCATGCCCTCGAAAAGCTATG-3′ (forward) and 5′-TGACGCATTGTTAAGGCTTCT-3′ (reverse); β-actin, 5′-AGAGGGAAATCGTGCGTGAC-3′ (forward) and 5′-AGGAGCCAGAGCAGTAATCTC-3′ (reverse); miR-7a stem loop, 5′-CTCAACTGGTGTCGTGGAGTCGGCAATTCAGTTGAGACAACAAA-3′, miR-7a forward, 5′-ACACTCCAGCTGGGTGGAAGACTAGTGATTT-3′; miRNA Universal Reverse, 5′-TGGTGTCGTGGAGTCG-3′; U6 Forward, 5′-CTCGCTTCGGCAGCACA-3′, U6 Reverse, 5′-AACGCTTCACGAATTTGCGT-3′.

### Western blot analysis

Cells were washed twice with ice-cold PBS after collection. Total proteins in cells were lysed with RIPA buffer (protein inhibitor included) for 40 min then centrifuged (12,000 rpm, 10 min, 4°C). The extracted proteins were separated using 12% SDS-PAGE and transferred to PVDF membranes (Millipore, Bedford, US). After that, the membranes were blocked in non-fat 5% milk at room temperature for 1 h then blotted with an appropriate primary antibody at 4°C overnight. After three washes in PBS containing 0.1% Tween 20, the membranes were incubated in a second antibody of mouse or rabbit specific for each primary antibody for 1 h at room temperature the following day. After three washes in PBS with 0.1% Tween 20, the blots were visualized using ECL detection reagents. Antibodies used for Western Blotting were as follows: anti-FADD (Abcam, ab108601), anti-FAK (BD Bioscience, 610088), anti-β-actin (ABGENT, AM1021B), anti-GAPDH (Santa Cruz biotechnology, L-3113) and anti-alpha-Tubulin (Epitomics, 2871-1). b-Actin, GAPDH, a-Tubulin served as loading controls in different experiments. Bands were quantified by Image J software.

### Scratch wound healing assay

After a 36 h period post-transfection with miRNA mimic/inhibitor, siRNA or vector, cells were seeded to 90% confluence in a six-well plate for overnight culture. The transfected cells were kept in serum-free medium for 12 h before the following morning a scratch was made with a 200 ml sterile pipette tip to create an open “wound”. Then the dislodged cells were removed by three washes with PBS, the rest adherent cells were still incubated in serum-free DMEM. Migration into the open wound was photographed at different time points until the scratch was nearly closed.

### Pulmonary metastasis model assay

C57BL/6 mice were divided into 4 groups (5 animals/group): pRK5, pRK5-FADD, pRK5-FADD+miR-7a mimic and pRK5-FADD+miR-7a inhibitor. B16F1 melanoma cells treated with pRK5 empty vector plasmids, pRK5-FADD plasmids, pRK5-FADD plasmids plus miR-7a mimic and pRK5-FADD plasmids plus miR-7a inhibitor, were collected and suspended in PBS. Then the treated B16F1 cells (6 × 10^5^ cells in 200 ml PBS per mouse) were injected into the tail vein of C57BL/6 mice. After 14 days, mice were sacrificed and lung tissues were excised. Surface lung tumor nodules were counted.

### Statistical analysis

Each experiment was repeated at least three times. The results were presented as the means ± SD for all values. The differences between groups were analyzed with Student's t-test; P-values indicated in figures are <0.05 (*), <0.01 (**), and <0.001 (***).

## Abbreviations

FADD: Fas-associated protein with death domain
FAK: Focal Adhesion Kinase
GAPDH: glyceraldehyde-3-phosphate dehydrogenase
MEF: mouse embryonic fibroblasts
NC: negative control
qRT-PCR: quantitative realtime PCR
PBSCDS: phosphate buffered saline
FBS: fetal bovine serum
DMEM: Dulbecco's modified Eagle's medium
